# Exploiting viral natural history for vaccine development

**DOI:** 10.1007/s00430-015-0406-1

**Published:** 2015-03-21

**Authors:** Peter A. Barry

**Affiliations:** Center for Comparative Medicine, University of California, Davis, One Shields Avenue, Davis, CA 95616-5270 USA

**Keywords:** Cytomegalovirus vaccine, Viral interleukin-10, Immune modulation, Viral natural history

## Abstract

The partial successes of the Phase 2 gB-based vaccine trials for HCMV highlight the very real likelihood that vaccine-mediated induction of antibodies that neutralize the fusion pathway of fibroblast infection is not sufficient as a singular strategy to confer protective efficacy against primary HCMV infection. Alternative strategies that serve as adjuncts to gB-based vaccines are likely required to target different aspects of the complex lifecycle of HCMV infection. There has been considerable recent interest in targeting the gH/gL/UL128/UL130/UL131 pentamer complex (gH/gL-PC) to neutralize the endocytic pathway of HCMV infection of epithelial and endothelial cells. Since both cell types are critical during primary mucosal infection, intrahost spread, and shedding of HCMV in an infected host, the gH/gL-PC represents a high-value target for vaccination to interrupt the HCMV lifecycle. The natural history of HCMV is exceedingly complex and incompletely resolved, and the protective efficacy generated by gH/gL-PC remains to be validated in clinical trials. Yet, there are salient aspects of its lifecycle that offer clues about how other novel vaccine strategies can be targeted to especially susceptible parts of the viral proteome to significantly disrupt HCMV’s ability to infect susceptible hosts. In particular, the protracted evolution of *Herpesvirales* has endowed HCMV with two remarkable properties of its natural history: (1) lifelong persistence within immune hosts that develop extraordinarily large antiviral immune responses and (2) the ability to reinfect those with prior immunity. The latter phenotype strongly implies that, if HCMV can overcome prior immunity to initiate a new infection, it is likely irrelevant whether prior immunity derives from prior infection or prior vaccination. Both phenotypes are unified by the extensive devotion of the HCMV coding repertoire (~50 %) to viral proteins that modulate host cell signaling, trafficking, activation, antigen presentation, and resistance to apoptosis. Collectively, these viral proteins are the likely reason for the high barrier to success for the 4-decade effort to design an HCMV vaccine, and they represent the viral proteins that make HCMV be the virus that it is. James Hanshaw wrote in 1971 that, based on a 15-year retrospective of congenital HCMV cases, “… *any thoughtful program designed at prevention or treatment deserves consideration*”. Drawing upon natural history data from the nonhuman primate model of HCMV persistence and pathogenesis, a “*thoughtful program*” is put forth that HCMV immune-modulating proteins should be considered as vaccine candidates.

## Introduction

Some of the earliest observations of CMV infections in nonhuman primates were remarkably prescient about contemporary CMV research occurring eight decades later (Table 1). Three years after it was demonstrated (1926) that the salivary gland agent of guinea pig was a virus that stimulated pathognomonic intracellular inclusions in infected cells, similar inclusion-bearing cells were observed in rhesus macaques acutely infected with poliovirus [1]. Inclusion-bearing cells were observed in the majority of study animals. The authors concluded that the intracellular lesions, phenotypically consistent with those described in guinea pigs, were unrelated to poliovirus infection. The authors further noted that cytomegalic cells were often associated with mixed lymphocytic infiltrates but sometimes were unaccompanied by any signs of a local inflammatory response. The occasional absence of inflammatory cells localized to a cytomegalic cell that we now recognize as one expressing the full complement of viral proteins is consistent with viral modulation of host immune responses in the microenvironment of that infected cell. These observations were extended 2 years later in 1931 by Covell [2] who concluded that the lesions, found in epithelial cells of multiple tissues, were not associated with clinical signs of infection and were “*caused by some virus of low virulence*”. Finally, Cowdry and Scott [3] noted that treatment of macaques with irradiated ergosterol, a sterol found in the cell membranes of fungi and yeasts, stimulated increased numbers of cytomegalic cells compared with untreated animals. The authors concluded that treatment with ergosterol “*may have activated or intensified a process already latent in the kidneys*” and that the “*association between persistence of inclusions and presence of active virus may be stressed*”.

**Table 1:** Early recognition of key features of CMV natural history in nonhuman primates

Phenotype	Year	Reference
Inclusion bodies	1929	[1]
Ubiquitous		
Immune modulation		
Low virulence	1931	[2]
Broad tissue tropism		
Reactivation from latency	1935	[3]
Persistence		

Thus, within 10 years of the recognition that a filterable agent of animals induced cytomegalic cells that were identical in description to those identified in infants who died shortly after birth [4, 5], the essence of CMV natural history was set forth. By the end of 1935, CMV was characterized as a ubiquitous virus with a broad tissue tropism that stimulates protective immune response in infected hosts, resulting in low pathogenic potential, yet persists in that same immune host, in part through establishment of and reactivation from latency, and in part through immune modulation.

This duality of CMV’s effect on the host immune system, stimulating immune responses that protect against clinical disease yet fail to clear persistent and active virus, represents something of a Gordian knot that requires thinking outside of the box to solve the underlying conundrum. Much as Winston Churchill recognized in 1939 that there was a key to predicting Russia’s actions (“*a riddle wrapped in an mystery inside an enigma*”), there, too, is a key to the seeming paradoxes of CMV natural history. For Churchill, the key was Russian national interests.

For CMV, the key is likely found within CMV and *Herpesvirales* evolution from a progenitor herpesvirus, which has been estimated to have arisen 200–400 M years ago [6, 7]. As obligate intracellular pathogens, physical, innate, and adaptive barriers to infection represent exceedingly high thresholds for a virus to enter a cell and subsume host machinery to generate progeny virions that can spread both within the infected host and between hosts. Given that herpesviruses infect countless vertebrate species, and at least one invertebrate species, the 200–400 M years of intervening time since the progenitor herpesvirus represents 200–400 M years of Darwinian selection, and that for every variant that arose, “*rejecting that which is bad, preserving and adding up all that is good; silently and insensibly working* [*at*] *the improvement of each organic being*” [8]. Put another way, an order of viruses that has evolved on a geologic timescale (since Devonian to Jurassic periods) has to be exceedingly good at what it does to still be around to infect us, let alone be fascinating topics of research.

While all herpesviruses establish and maintain lifelong persistence within infected hosts, the virus–host relationship between HCMV and infected humans appears to be unique among the herpesviruses, and definitely unlike any other characterized pathogen–host interaction. The pioneering study of Sylwester et al. [9], in which the T cell response to the HCMV proteome was quantified, revealed that there is an extraordinarily large devotion of the T cell repertoire (~10 % of both memory CD4^+^ and CD8^+^ T cells) to a single pathogen. This massive T cell response is undoubtedly essential for ensuring that HCMV is a virus with low pathogenic potential in immune competent hosts. This interpretation is substantiated by the fact that impairment of immune functionality, such as that following iatrogenic immunosuppression or HIV–AIDS, is associated with dramatically increased risks of HCMV morbidity and mortality. The T cell response to HCMV may have been the result of an evolutionary arms race with the coding sophistication within the HCMV genome. Between 56 and 72 % of the Towne and AD169, open reading frames (ORF) can be individually deleted, respectively, without impairing replication in fibroblasts [10, 11]. The CMV family has evolved redundant and/or overlapping functions for many viral proteins, thus enabling the deletion of one ORF without compromising replicative fitness within fibroblasts. However, another interpretation of ORF being dispensable for replication in cultured cells is that they are *indispensable* for viral infection within the host. While functions for many ORF remain to be defined, those ORF, which have been characterized, broadly target multiple aspects of innate and adaptive effector functions [12, 13], and the reasonable conclusion is that HCMV is “armed for bear” when it comes to countering almost every aspect of host immunity. And these viral immune-modulating proteins have conferred a remarkable ability on HCMV.

Whereas viruses such as HIV and influenza undergo genetic drift as a mechanism for emergence of immune escape variants, this is not a strategy employed by HCMV to counter host effector functions. It does not appear that there have been descriptions of immune escape variants for HCMV. This stands in marked contrast to the occasionally rapid emergence of drug-resistant variants following antiviral treatments. The simplest interpretation is that HCMV never had to evolve other strategies to counter anti-HCMV drug regimens that are very recent in the context of herpesvirus evolution. In contrast, herpesviruses in general, and CMV in particular, evolved in the context of evolutionarily ancient innate adaptive immune systems, and in the context of the more recent adaptive immune systems and an extensive array of haplotypes. Taken together, the arsenal of HCMV-encoded immune-modulating proteins obviated the need to resort to genetic drift to counter host immunity. By extension then, this class of viral proteins defines the essence of HCMV natural history, and abrogation of their functions should significantly impair HCMV’s ability to successfully complete in life cycle within an infected host. Accordingly, the hypothesis is presented that immune-modulating proteins of HCMV should be considered as vaccine targets to serve as adjuncts to the current paradigms of neutralizing viral attachment to susceptible cells. This review will primarily draw upon work in the nonhuman primate model of HCMV persistence and pathogenesis involving infection of rhesus macaques (*Macaca mulatta*) with rhesus CMV (RhCMV), together with notable findings of HCMV that highlight the importance of immune modulation in primate CMV natural history.

## Manipulation of the IL-10 signaling pathway

A central feature of microbial pathogenesis, including CMV, is that pathogens have evolved a myriad of virulence strategies involving attenuation, exploitation, and/or cooptation of normally protective immune functions to enable the completion of the pathogen’s life cycle within the host and dissemination to the next susceptible host. While there are pathogen-specific mechanisms for altering pathogen–host interactions, there are also common immune-modulating pathways shared among evolutionarily diverse pathogens. In particular, a large number of viruses, protozoa, pathogenic and commensal bacteria, helminths, and fungi all utilize manipulation of the interleukin-10 (IL-10) signaling pathway as part of microbial natural history [14]. Given that IL-10 is a master immune regulator with pleiotropic effects on multiple cell types [15], it is no surprise that pathogens target this singular pathway to subvert protective effector functions. One emerging theme is that pathogens and commensal bacteria have coopted IL-10 signaling to enable states of immune tolerance, immune suppression, and immune evasion, strategies that are particularly salient for those pathogens that establish lifelong persistence [16, 17].

The vast majority of pathogens that exploit the IL-10 signaling pathways have evolved strategies that upregulate IL-10 expression by host cells, although the precise mechanism by how this occurs remains incompletely resolved. However, certain members of *Herpesviridae* and *Poxviridae* transduced cellular IL-10 (cIL-10) genes at some point in their evolution and now express the viral IL-10 (vIL-10) orthologs in the context of viral infection [18]. [NOTE: vIL-10 is used as a generic term for the cIL-10 ortholog encoded within viral genomes.] Identification of a multiply sliced vIL-10 transcript in RhCMV (rhcmvIL-10) was a serendipitous discovery. Based on the presence of the rhcmvIL-10 gene within the RhCMV genome, reanalysis of the AD169 coding content revealed that the original annotation for potential ORF within the AD169 sequence [19] missed the presence of the HCMV-encoded cmvIL-10 (UL111a) due to the sizes of the three exons of cmvIL-10 falling below the 100 amino acid threshold for identifying ORF [20]. Both HCMV- and RhCMV-encoded vIL-10 orthologs are about as divergent from the cIL-10 proteins of their host (27 and 25 % identity, respectively), as they are from each other (31 % identity). Sequencing of the vIL-10 gene from multiple strains indicates that the vIL-10 protein is very stable for intraspecies comparisons of HCMV or RhCMV strains [21]. cmvIL-10 and rhcmvIL-10 do not represent a hyper-variable region within the HCMV and RhCMV genomes. The extensive drift from the cIL-10 proteins of their respective host species strongly alludes to the functional dominance of this viral protein in HCMV and RhCMV natural histories.

Whatever selective pressures there were that might account for the current sequence divergence of cmvIL-10 and rhcmvIL-10 from cIL-10, the end result is that what was once a host “self”, non-immunogenic protein expressed during progenitor primate CMV infection has diverged to become a highly immunogenic protein. RhCMV vIL-10 stimulates robust titers of both rhcmvIL-10-binding and rhcmvIL-10-neutralizing antibodies in RhCMV-infected macaques [22]. Antibody responses to cmvIL-10 have also been reported in HCMV-infected humans [23]. One simple interpretation is that the positive selective advantage conferred by retaining only 25–27 % sequence identity with the host cIL-10 must exceed the negative selection resulting from an increased immunogenic profile. To date, there is no evidence that cmvIL-10 drift has resulted in acquisition of new functionalities beyond those of cIL-10 [18]. It should be emphasized that this refers to the full-length cmvIL-10 protein. Notably, an incompletely spliced variant of vIL-10 found during latent infection (LAcmv-IL-10) does have novel functionalities that are independent of binding to the high-affinity IL-10R1 receptor [13, 18, 24]. The strongest clue to what might account for the extensive genetic drift of cmvIL-10 and rhcmvIL-10 relates to the relative binding affinities of cmvIL-10 and cIL-10 for the cellular IL-10R1 receptor. cmvIL-10 has higher binding affinity for IL-10R1 than cIL-10, and one possibility is that sequence differences between HCMV and RhCMV vIL-10 proteins were compensatory changes to differences between human and macaque IL-10R1 proteins [25]. One hypothesis that remains to be tested is that the ability of vIL-10 to outcompete cIL-10 for IL-10R1 confers greater fitness to HCMV and RhCMV than the negative consequences of drifting to an immunogenic form of the protein [21, 25].

Emerging evidence for the vIL-10 proteins of both HCMV and RhCMV highlights that vIL-10 broadly targets innate immune cells. Based on in vitro assays following exposure of macrophages (Mϕ), peripheral blood mononuclear cells (PBMC), monocyte-derived dendritic cells (MDC), and plasmacytoid dendritic cells (PDC) to vIL-10 [18, 27–32], expression of vIL-10 in vivo should have pleiotropic immunosuppressive effects on multiple cell types (Table 2). It is especially notable that the functionality of vIL-10 is directed at key innate cell types, particularly monocytes/Mϕ, MDC, and PDC, that are links between the innate and adaptive immune responses. vIL-10-mediated modulation of the functionality of these cells should have the effect of directly influencing the quality of the adaptive antiviral immune responses.

**Table 2:** Functional activity of vIL-10 on lymphoid cells in vitro

Cell type(s)	Phenotype	Ref.
Monocytes	↑ Differentiation into inflammatory macrophages	[26]
	↓ HLA-DR	
	↑ CD64, CCR5	
PBMC	↓ Mitogen-stimulated proliferation, MHC-I and –II	[27, 28]
	↓ IFN-γ	
Monocytes	↓ IL-6, IL-1α, GM-CSF, TNF-α; MHC class I and II	
	↑ HLA-G	
MDC	↓ TNF-α, IL-6 IL-12, Bcl-2, Bcl-XL	[29, 30]
	↑ cIL-10	
PDC	↓ INF-α, IFN-β	[31]

Based on the functionalities of vIL-10 on the different cell types in Table 2, it is proposed that vIL-10, likely in conjunction with other viral proteins, exerts a viral adjuvant-like effect on long-term antiviral immunity. While it remains to be determined whether other HCMV proteins exert comparable effects on innate cells, the hypothesis leads to a model whereby lifelong persistence is initiated at the earliest virus–host interactions following primary mucosal infection [16, 26]. Support for this comes from inoculation of naïve rhesus macaques with the 68-1 strain of RhCMV, a fibroblast-adapted strain, and an engineered variant of 68-1 in which the rhcmvIL-10 gene had been deleted (RhCMV ΔvIL-10) [26]. Prominent distinctions were observed in both the acute and long-term immune responses, including the extent and cell composition of the cellular infiltrate at the site of subcutaneous (SC) inoculation, and the early and long-term anti-RhCMV adaptive immune responses. Based on this result, a converse approach, i.e., vaccination against rhcmvIL-10, was evaluated to determine to what extent rhcmvIL-10 influences the virus–infected host relationship.

## Vaccination against rhcmvIL-10

As noted, rhcmvIL-10 is strongly immunogenic when expressed in the context of RhCMV infection [22], establishing a precedent to immunize macaques with a recombinant form of rhcmvIL-10. However, vaccination of naïve animals with fully immunosuppressive vIL-10 would likely stimulate immune responses very distinct, and likely undesirable, from those induced by vaccination with a non-functional form of rhcmvIL-10. To maximize immunogenicity of rhcmvIL-10, minimal alterations were engineered into rhcmvIL-10 (2 amino acids) to create a biologically inactive form of the protein, by abrogating binding to IL10R1 [22, 33]. The specific amino acids that were changed were based on the by crystal structures of cIL-10/IL-10R1 and cmvIL-10/IL-10R1 complexes [25]. Immunization of RhCMV-uninfected macaques by a combined DNA prime/protein boost immunization strategy stimulated both rhcmvIL-10-binding and rhcmvIL-10-neutralizing antibodies that did not exhibit any cross-reactivity with rhesus macaque cIL-10 [34]. Vaccinated animals were subsequently challenged with the epitheliotropic UCD59 strain of RhCMV and evaluated for molecular and immunological parameters of infection. The results demonstrated that vaccination against a single RhCMV immune-modulating protein, rhcmvIL-10, significantly altered viral parameters of challenge infection, including reductions locally at the site of SC inoculation, and in the frequency and magnitude of RhCMV shedding in saliva and urine. This proof-of-principal study demonstrated for the first time for CMV that vaccine-induced neutralization of the functionality of a single immune-modulating protein (rhcmvIL-10) confers protective efficacy in the absence of vaccine-induced antibodies that neutralize viral attachment to susceptible cells. Extrapolating from this result in macaques to human, vaccination against cmvIL-10 should have comparable efficacy in protecting against primary HCMV challenge and the establishment of a persistent infection. In addition, vaccination could also protect against the maintenance of persistence, based on recent studies on cmvIL-10 expression during latency.

## IL-10 signaling during latency

It is well established that HCMV establishes a latent infection in CD34^+^ hematopoietic progenitor cells [35], and recent studies indicate that persistence is maintained, in part, through HCMV-induced generation of CD4^+^ T cells that secrete cIL-10 in response to HCMV antigens. Two hallmarks of latency in these cells are an extremely restricted pattern of viral gene expression and the absence of infectious virion production [36, 37]. The viral transcriptome defining latently infected CD34^+^ cells includes cmv-IL-10 [24] and LAcmv-IL-10 [38], the aberrantly spliced transcript that results in a truncated, but biologically functional, form of cmvIL-10 that does not engage IL-10R1 [39, 40]. Expression of LAcmv-IL-10 in latently infected CD34^+^ cells stimulates expression of cIL-10 in a mechanism that appears to involve suppressed expression of the microRNA hsa-miR-92a [38]. The authors note that expression of LAcmv-IL-10 in a paracrine and/or autocrine fashion “*modulates the microenvironment of the infected cell, leading to evasion of the immune system*”. They further speculate that LAcmv-IL-10 represents a potential therapeutic target since LAcmv-IL-10 is very distinct in sequence from cIL-10. The limited expression of the HCMV proteome in latently infected cells also elicits a very specific effect on the type of antigen-specific T cells. Expression of cmvIL-10, like that of LUNA, UL138, and US28, stimulates antigen-specific CD4^+^ T cells that secrete cIL-10 in response to antigen exposure, leading to the suggestion that “*HCMV skews the T cell responses to latency*-*associated antigens to one that is overall suppressive in order to sustain latent carriage* in vivo” [41]. Viral induction of cIL-10 signaling parallels MCMV-mediated induction of CD4^+^ T cells secreting cIL-10 [42], lending support to the hypothesis that disruption of the cIL-10 signaling pathway in persistent infections may be one way of altering the virus–host homeostasis in persistently infected hosts to a state favoring host immune-mediated clearance of virally infected cells [16]. The mechanisms that HCMV appears to utilize in skewing host immune responses during a persistent infection may also be operative during reinfection.

It is now accepted that HCMV can reinfect those with prior immunity, which assumes clinical importance in the context of congenital infections in populations with near universal HCMV seroprevalence [43–47]. Despite the extraordinarily large T cell response devoted to HCMV antigens and the broad neutralizing responses against multiple HCMV glycoproteins involved in attachment and entry [9, 48–52], HCMV can reinfect immunocompetent individuals with preexisting immunity to HCMV. At this point, there is no evidence indicating that reinfection involves selection for distinctly antigenically variant strains of HCMV, which leaves the hypothesis that modulation of host immunity during reinfection enables the reinfecting strain to disseminate the mucosal surface to distal sites, particularly the maternal/fetal interface. While studies investigating the role the vIL-10 may play during HCMV and RhCMV reinfection are ongoing, results from the rhesus model unequivocally demonstrate that modulation of host immunity is essential for RhCMV reinfection.

## Attenuation of host immunity during RhCMV reinfection

Rhesus CMV (RhCMV) encodes sequence and functional orthologs of HCMV US2, 3, 6, and 11, and like their HCMV counterparts, the RhCMV proteins disrupt antigen presentation by major histocompatibility complex (MHC) class I [53]. The RhCMV orthologs of US2-11 are not required for the establishment of a persistent infection following SC inoculation of RhCMV-uninfected macaques with an engineered variant of 68-1, in which the RhCMV US2-11 orthologs were deleted, and the SIV gag gene was coexpressed [54]. Animals developed long-term RhCMV- and Gag-specific T cell responses, and input virus could be recovered from the urine of inoculated animals almost 2 years after primary inoculation. However, when animals with preexisting immunity to RhCMV were inoculated with the same US2-11-deleted variant (and co-expressing SIV gag), there was absolutely no evidence of reinfection. Animals did not develop Gag-specific T cell immune responses, and the input virus was never recovered from the urine. In contrast, inoculation of immune animals with the parental construct expressing US2-11 efficiently resulted in reinfection. This study emphasizes that in this model, antigenic variation is not the critical factor for reinfection. Rather, attenuation of host antiviral immune responses, in this case through disruption of MHC class I antigen presentation, is essential for reinfection.

## Summary

Taken as a whole, accumulating data on the immune-modulating proteins of HCMV and RhCMV highlight their central role during all phases of the infectious cycle within an infected host, beginning with the immediate virus-mediated modulation of host immune responses at the mucosal surface of a naïve host, to maintenance of an immune suppressive environment in latently infection CD34^+^ cells, to suppression of effector/memory responses during reinfection of an immune host. HCMV has had eons to optimize an orchestrated symphony of viral functions that target almost every aspect of innate and adaptive immunity that we as hosts can throw against it. Because of this, HCMV immune modulation represents an opportunity to leverage some of these qualities to change human health. The dramatic and extremely promising results obtained using RhCMV-based vaccine vectors to protect against virulent SIV challenge are possible only because of RhCMV-encoded immune modulatory proteins [55–57]. These functions enable reinfection of and long-term persistence in immune animals, facilitating long-term, non-classical CD8^+^ T cell responses that can protect against SIV challenge years after immunization. However, this armada of immune-modulating functions constitutes a significant challenge to overcome in designing vaccine strategies that prevent primary and non-primary infections. Taking Hanshaw’s declaration for “*any thoughtful program designed at prevention*” [58] to heart, the weight of evidence points to some of the viral immune-modulating proteins as especially susceptible targets of the viral proteome for vaccine strategies.
